# Brain MRI features of methylmalonic acidemia in children: the relationship between neuropsychological scores and MRI findings

**DOI:** 10.1038/s41598-020-70113-y

**Published:** 2020-08-04

**Authors:** Linfeng Yang, Bin Guo, Xue Li, Xiangyu Liu, Xinhong Wei, Lingfei Guo

**Affiliations:** 1 Jinan Maternal and Child Care Hospital, Jian-Guo Xiao Jing-San Road No. 2, Jinan, 250001 Shandong People’s Republic of China; 2 Department of MRI Room, Shandong Medical Imaging Research Institute, Cheeloo College of Medicine, Shandong University, Jing-wu Road No. 324, Jinan, 250021 Shandong People’s Republic of China

**Keywords:** Paediatric research, Metabolic disorders, Neurological disorders, Neuroscience

## Abstract

Methylmalonic acidemia (MMA) is a severe, heterogeneous disorder of methylmalonate and cobalamin (cbl; vitamin B12) metabolism with a poor prognosis that can cause brain damage. Identifying the magnetic resonance imaging (MRI) findings of MMA might help to make accurate diagnoses earlier in the disease course and exploring the relationship between neuropsychological scores and MRI findings, when therapy is more effective and to improve therapeutic efficacy. Cerebral MRI studies from 37 children with MMA were evaluated by a neuroradiologist. Clinical and imaging data were collected from each patient. All tests were performed during routine investigations and in accordance with the ethical principles of the Declaration of Helsinki. Informed consent was obtained from the guardians of all patients for inclusion in the study. The most common and significant findings were periventricular white matter changes (78.4%), ventricular dilation (29.7%) and cerebral atrophy (40.5%). According to the developmental quotient, the 37 patients were divided into the normal intelligence subgroup (NI, developmental quotient ≥ 85) and the low intelligence subgroup (LI, developmental quotient < 85). The incidence of corpus callosal thinning, cortical atrophy, subcortical white matter changes, and ventricular dilation (grades 0–3) was significantly higher in the LI subgroup than in the NI subgroup (P < 0.05). The incidence of no-mild and moderate-severe ventricular dilation was significantly higher in the LI subgroup than in the NI subgroup (P < 0.05). Ventricular dilatation, cerebral atrophy, white matter changes, and corpus callosal thinning are the main MRI abnormalities in MMA patients, and these manifestations are significantly correlated with delayed development in children.

## Introduction

Methylmalonic acidemia (MMA) is a lethal, severe, heterogeneous disorder of methylmalonate and cobalamin (cbl; vitamin B12) metabolism with a poor prognosis, as noted in 1967 in the first report of this disease^1^. Defects in methylmalonyl-CoA mutase (MCM) or its coenzyme, cobalamin, lead to the accumulation of methylmalonic acid, which is characteristic of methylmalonic acidemia (MMA)^2–4^.

According to the type of enzyme deficiency, there are two categories of MMA: MCM deficiency (also referred to as the mutant type (mut type)) and coenzyme cobalamin metabolism disorder (or the cobalamin type). A mutation in the gene encoding MCM causes the mut type; this mutation alters the activity of the allosteric enzyme. The cobalamin type is caused by congenital defects in the synthesis, activation or transport of cobalamin (the allosteric coenzyme), which affect the function of MCM by disrupting the synthesis of its coenzyme deoxyadenosylcobalamin. Accumulation of homocysteine and occurs in isolation or combined with MMA mutase deficiency results from deficiency of this enzyme defects of intracellular cobalamin metabolism as well as in nutritional deficiency or disturbed uptake or transport of vitamin B12. The cobalamin type can be subdivided into six subtypes (cblA, cblB, cblC, cblD, cblF, and cblH). The mut type and the cblA, cblB, and cblH deficiency types present as MMA alone and are classified as isolated MMA; the cblC, cblD, and cblF deficiency types are classified as MMA combined with homocysteinemia^5,6^, MMA combined with homocysteinemia is the main biochemical type of MMA in China, accounting for 60–80% of cases^7^, and cblC is the most common subtype^8,9^.

MMA often causes damage to multiple body systems, especially the central nervous system^10^. Although the incidence is low, the mortality and disability rates are very high^11^. This disease has diverse and nonspecific clinical manifestations,the main manifestations are feeding difficulties, intellectual disabilities, ataxia, abnormal muscle tone, convulsions, epilepsy, and lethargy^12,13^. As a result, patients often need to undergo many tests before a correct diagnosis can be made. Symptoms of neurological damage are common in patients with MMA, and brain imaging findings are often used to rule out congenital or acquired cerebral abnormalities. Magnetic resonance imaging (MRI) causes no radiation damage and is suitable for brain examinations in children. This modality is increasingly used in neuroimaging studies of genetic metabolic diseases. Identifying the magnetic resonance imaging (MRI) findings of MMA might help to make accurate diagnoses earlier in the disease course and exploring the relationship between neuropsychological scores and MRI findings, when therapy is more effective and to improve therapeutic efficacy^14^.

## Materials and methods

### Patients and diagnosis

This study was approved by the Institutional Review Board (IRB) of Jinan Maternal and Child Care Hospital, Shandong. Informed consent was obtained from the guardians of all patients for inclusion in the study. Thirty-seven patients were diagnosed with MMA according to the MMA diagnostic criteria and were included in the study. The standard protocol for the diagnosis of MMA was increased plasma methylmalonyl carnitine or increased urine methylmalonic acid, as detected by gas chromatography-mass spectrometry (GC/MS) and the propionate incorporation test. The levels of propionyl carnitine (C3), C3/acetylcarnitine (C2) were measured by tandem mass spectrometry methionine in dried blood spots. To further confirm the diagnosis, the levels of organic acids in urine were measured with GC/MS in patients with suspected MMA. In addition, the concentration of total plasma homocysteine (tHcy) and hyperammonemia was measured^15^, detailed genetic data and experimental data in Supplementary materials. Nineteen patients underwent genetic newborn screening for neonatal genetic diseases after birth, and 18 patients underwent blood and genetic screening after exhibiting suspicious symptoms (SS). MRI examination and neuropsychological tests were performed on these patients before treatment. The neuropsychological test used a pediatric examination table of neuropsychological development for 0- to 6-year-old patients (CNBSR 2016). All tests were performed as a part of routine clinical and biochemical investigations and were performed in accordance with the ethical principles of the Declaration of Helsinki.

### Demographic and clinical data acquisition

We gathered clinical data, laboratory test results, gene type, including age at the time of MRI and symptoms for each patient. These data were obtained from the genetic metabolic disease clinic, and medical history was obtained from the guardian if needed. Cerebral MRI scans of the patients were retrieved, and those who had no previous cerebral MRI were excluded. We identified 37 studies from 70 patients who had been diagnosed with genetic metabolic diseases. The MRI findings of these 37 patients were reviewed, including the findings of 27 (73.0%) males and 10 (27%) females (see Table 1). Thirty-seven patients received standardized treatment. After vitamin B12 stress test, vitamin B12 is effective in patients with cblC, the patients with mut did not respond. The patients with cblC were treated with hydroxocobalamin (OHCbl) (1 mg/day, intramuscular injection), betaine (100–500 mg/kg/day, oral administration), folic acid(5–0 mg/day, oral administration),vitamin B6 (10–30 mg/day, oral administration), sodium benzoate(150–250 mg/kg/day, improve hyperammonemia), levocarnitine (50 mg/kg/day, oral administration), low protein and high-energy diet to reduce the accumulation of toxic metabolites. The patients with mut were treated with Vitamin B6 (10–30 mg/day, oral administration),Sodium benzoate(150-250 mg/kg/day, improve hyperammonemia), L-carnitine (50–100 mg/kg/d, orally or intramuscular injection) and were restricted natural protein. Some patients with motor system damage need to carry out sensory, motor function rehabilitation training and language cognitive ability training in order to facilitate the growth and development of patients.

**Table 1:** Demographic data and clinical findings in 37 children with MMA.

Patient	Gender	AP (m)	AI (m)	Gene type #	The group of by clinical screening*	First presenting symptom	0–6-year-old pediatric examination table of neuropsychological development						
							Big activity sport	Fine sport	Adaptive capacity	Language capacity	Social behavior	Development quotient	Developmental quotient evaluation
1	M	18	19	cbIC (MMA-h)	SS	Developmental regression; hypotonia; respiratory distress	33.1	24.8	0	33.1	0	18.2	Low
2	F	23	23	cbIC (MMA-h)	SS	Psychomotor delay	81	71	65	55	71	68.6	Low
3	F	6	7	cbIC (MMA-h)	SS	Growth retardation	58.8	49	39.2	49	53.9	50	Low
4	M	6	6	cbIC (MMA-h)	SS	Growth retardation; seizure	83.3	90.9	75.8	90.9	90.9	86.4	Normal
5	M	1	1	cbIC (MMA-h)	SS	Respiratory distress	86	89	88	90	91	88.8	Normal
6	M	30	32	cbIC (MMA-h)	SS	Coma; recurrent vomiting	85.5	87	90	93	89.5	89	Normal
7	M	21	24	cbIC (MMA-h)	SS	Lethargy	90	94	86	91.5	94	91.1	Normal
8	F	47	48	mut(i-MMA)	SS	Developmental regression	87	90	91	93	88	89.8	Normal
9	M	60	60	mut(i-MMA)	NS	Screening	93.2	93.2	93.2	103	93.2	95	Normal
10	F	41	41	cbIC (MMA-h)	SS	Skeletal abnormality; lethargy	42.4	72.7	69.6	72.7	90.9	69	Low
11	M	19	19	cbIC (MMA-h)	SS	Recurrent vomiting	70	69.5	72	73	71	71.1	Lower
12	M	35	35	cbIC (MMA-h)	NS	Screening; lethargy	109.2	70.2	98.8	88.4	88.4	91	Normal
13	M	10	10	cbIC (MMA-h)	SS	Developmental regression	97.8	73.4	73.4	73.4	59.8	75.5	Lower
14	M	10	11	cbIC (MMA-h)	NS	Screening	69	103.4	107.8	94.8	94.8	94	Normal
15	M	0.5	0.5	cbIC (MMA-h)	NS	Screening; emesis psychomotor delay	89	80	81	82	79	82.2	Lower
16	M	5	6	cbIC (MMA-h)	NS	Screening	70.3	85.9	93.8	93.8	85.9	85.9	Normal
17	M	3	4	cbIC (MMA-h)	NS	Screening; poor feeding	81.4	69.8	87.2	69.8	69.8	75.6	Lower
18	F	1.1	1.5	cbIC (MMA-h)	NS	Screening	82	65.6	69.7	57.4	73.8	69.7	Low
19	M	1.0	1.5	cbIC (MMA-h)	NS	Screening	75.9	75.9	71.4	53.6	71.4	69.6	Low
20	M	1	1	cbIC (MMA-h)	NS	Screening	108	85.2	108	68.2	90.9	92	Normal
21	M	0.4	0.5	cbIC (MMA-h)	NS	Screening	80	78	81	76	83	79.6	Lower
22	M	4	5	cbIC (MMA-h)	NS	Screening	119.2	72.8	79.5	79.5	69.5	84.0	Lower
23	F	5	5	cbIC (MMA-h)	NS	Screening	85.9	82	82	85.9	78.1	82.8	Lower
24	M	3	3	cbIC (MMA-h)	SS	Poor feeding; convulsion	62.5	80.4	80.4	71.4	89.3	76.8	Lower
25	M	4.5	5	mut(i-MMA)	NS	Screening	63.4	77.5	77.5	84.5	77.5	76.1	Lower
26	F	5	6	cbIC (MMA-h)	NS	Screening	67	93	56.8	56.8	82	70	Lower
27	M	7	9	cbIC (MMA-h)	SS	Seizure	84.4	61.7	80.2	74.1	61.7	72.8	Lower
28	M	2.6	2.9	cbIC (MMA-h)	SS	Recurrent vomiting	36	38	33	40	37	36,8	Low
29	M	3	4	cbIC (MMA-h)	NS	Screening; Seizure	64.9	32.5	45.5	64.9	51.9	51.9	Low
30	M	24	26	cbIC (MMA-h)	SS	Developmental regression	29	30	33	32	29	30.6	Low
31	M	4	5	cbIC (MMA-h)	NS	Screening	83.3	76.4	90.3	83.3	83.3	83.3	Lower
32	M	58	60	cbIC (MMA-h)	SS	Agitation	89	91	93	85	82	88	Normal
33	F	54	55	cbIC (MMA-h)	SS	Psychomotor delay	46.6	32.9	43.9	38.4	41.1	40.6	Low
34	M	11	13	cbIC (MMA-h)	NS	Screening	77.5	77.5	81.4	73.6	77.5	77.5	Lower
35	M	7.1	7.7	cbIC (MMA-h)	NS	Screening	40	39	38	37	41	39	Low
36	F	11	12	cbIC (MMA-h)	NS	Screening; agitation	88.4	73.2	79.3	61	64	73.2	Lower
37	F	70	72	cbIC (MMA-h)	SS	Microcephalus	24.3	28.4	18.2	20.3	20.3	22.3	Low

*AP* age at presentation, *AI* age at the time of imaging, *m* months, *,i-MMA* isolated MMA, M*MA-h* MMA combined with homocysteinemia.

*NS stands for the patients were genetically newborn screening for neonatal genetic diseases after birth, SS stands for the patients received blood and genetic screening after suspected symptoms. Developmental quotient evaluation: normal, 85–++4; lower, 70–84; low,≤69.

### MRI parameters and image interpretation

Patient imaging data were obtained using a 1.5-T MR scanner (Achieva, Philips Medical Systems) and a 16-channel phased-array head coil with T2-weighted imaging (T2WI) (repetition time (TR): 2,100 ms; echo time (TE): 100 ms), T1-weighted imaging (T1WI) (TR: 568 ms; TE: 15 ms), T2-weighted fluid-attenuated inversion recovery (T2-FLAIR) (TR: 700 ms; TE: 115 ms), and diffusion-weighted imaging (DWI) (TR: 3,090 ms; TE: 99 ms; b = 0 to 1,000) sequences with a field of view (FOV) of 230 × 190, slice thickness of 5 mm, gap of 0.5 mm, axial scanning, and sagittal T2WI (TR: 2,100 ms; TE: 90 ms) sections. The scan time was approximately 10 min.

Each image was evaluated by a senior pediatric radiologist who was unaware of the diagnosis. There are several reported scoring schemes for the severity of cortical atrophy and ventricular dilation, including the semiquantitative scale devised by Yue et al.^16^ and the age-related white matter changes (ARWMC) rating scale established by Wahlund LO^17^. After referencing the evaluation criteria proposed in the above two documents, the findings were scored as mild, moderate or severe based on a gross visual assessment and simplified scheme by the pediatric radiologist. We studied the findings in two different subgroups of MMA patients identified by newborn screening (NS) and MMA patients identified by suspected symptoms (SS): the normal intelligence (NI) subgroup and the low intelligence (LI) subgroup.

### Statistical analysis

Statistical analysis was performed using the Statistical Package for the Social Sciences (Version 21.0 for Windows; SPSS, Chicago, Ill)^18^ .*P* value of < 0.05 was considered to be statistically significant. Before performing individual analyses, the distributions of data sets were checked for normality. The measurement data were expressed as mean (standard) deviation, and the counting data as n (%). Chi-square test was used to compare the differences of image features between different groups. Considering that all variables in correlation analysis are classified variables or grade variables, Kendall coefficient is used for correlation analysis.

## Results

Among 37 patients (27 males and 10 females), SS of MMA were found in 18 patients, and 19 patients underwent genetic screening after birth for genetic metabolic diseases (mean age, 16.51 ± 19.52 months; age range, 0.5 to 54 months; age at the time of MRI, 17.35 ± 19.72 months; age range, 0.5 to 60 months). Patients with SS were diagnosed by MRI or were screened within no more than one month. These patients’ symptoms included seizure, developmental regression, hypotonia, psychomotor delay, recurrent vomiting, respiratory distress, agitation, coma, lethargy, growth retardation, and poor feeding. According to the table of neuropsychological development used in the examination of 0- to 6-year-old patients (CNBSR 2016), children who underwent the neuropsychological test were assigned a developmental quotient and divided into the normal developmental quotient and low developmental quotient subgroups. The classification standards were as follows: ≥ 130, excellent; 115–129, intelligent; 84–114, normal; 70–84, lower; ≤ 69, low. Scores ≥ 84 were considered normal, and scores < 84 were considered low. Among the 37 patients, the mean developmental quotient was 71.38 ± 20.83 (range, 18.2–94); 11 patients (29.7%) had a normal developmental quotient, and 26 patients (61.3%) had a low developmental quotient, as shown in Table 1.

**Figure 1:**
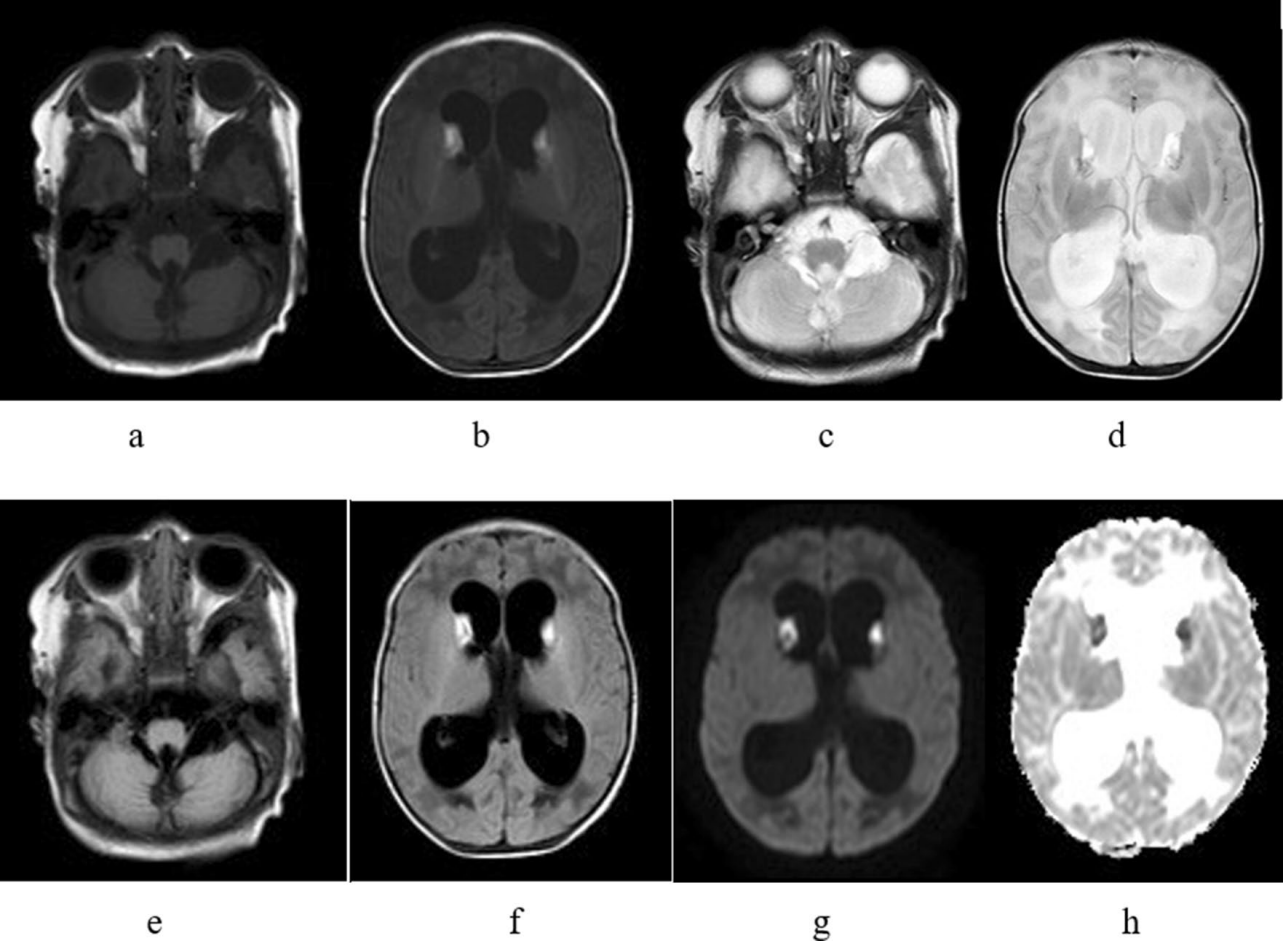
**(a-h)** **(b, d, f, e, h)**; hydrocephalus with small subcerebellar vermis, consistent with the Dandy–Walker variant (patient no. 19. MRI showing T1WI, T2WI, T2WI-FLAIR and DWI hyperintensity in the bilateral caudate nuclei and hypointensity in the corresponding ADC images, consistent with hemorrhage **a, c, e**).

The MRI findings were ventricular dilation, cortical atrophy (sulcal widening), corpus callosal thinning, brainstem thinning, subcortical white matter changes, periventricular white matter changes and signal changes consistent with focal infarct. The most common findings were periventricular white matter changes (78.4%), ventricular dilation (29.7%) and cerebral atrophy (40.5%), which were the most significant and severe imaging manifestations (see Table 2 for details). Symmetrical signal abnormalities were found in the bilateral caudate nuclei of one patient, as shown in Fig. 1.

**Table 2:** The MRI findings in 37 children with MMA.

Patient	The group of by clinical screening	Cerebral sulcus widened	Cerebellar atrophy	Brainstem thinning	Corpus callosum thinning	Cortical atrophy (sulcal widening)	Ventricular dilation (0–3)	Subcortical white matter change	Periventricular white matter change	Internal capsule change	Signal change consistent with focal infarct	Abnormal signal in basal ganglia
1	SS	−	−	−	+	−	Severe	Frontal	−	−	−	−
2	SS	−	−	−	−	−	−	−	Mild	−	−	−
3	SS	+	+	−	+	+	Severe	−	Mild	−	−	−
4	SS	+	+	−	−	+	−	−	−	−	−	−
5	SS	+	−	−	−	−	−	−	Mild	−	−	−
6	SS	−	−	−	−	−	−	−	Mild	−	−	−
7	SS	+	−	−	−	+	−	−	Mild	−	−	−
8	SS	−	−	+	−	−	−	−	Mild	−	−	−
9	NS	−	−	−	−	−	−	−	Mild	−	−	−
10	SS	+	+	−	+	+	Moderate	Frontal	Mild	+	−	−
11	SS	+	−	+	+	+	−	Frontal and parietal	Mild	−	−	−
12	NS	−	−	−	−	−	−	−	Mild	−	−	−
13	SS	−	−	−	+	−	−	Parietal	Mild	−	−	−
14	NS	−	−	−	−	−	−	Parietal	Mild	−	−	−
15	NS	−	−	−	−	−	−	Diffuse	Mild	+	−	−
16	NS	+	−	−	−	−	−	−	−	−	−	−
17	NS	+	+	−	+	+	Mild	−	−	−	−	−
18	NS	−	−	−	−	−	−	−	Mild	−	−	−
19	NS	+	−	−	+	−	Severe	Parietal	Moderate	−	+	+
20	NS	−	−	−	−	−	−	−	Mild	−	+	−
21	NS	−	−	−	−	−	−	−	Mild	−	−	−
22	NS	−	−	−	−	−	−	−	Mild	−	−	−
23	NS	−	−	−	+	−	−	−	Mild	+	−	−
24	SS	−	−	−	−	−	−	−	Mild	−	−	−
25	NS	−	−	−	−	−	−	Parietal	Mild	−	−	−
26	NS	+	−	−	+	+	Mild	−	−	−	−	−
27	SS	+	−	−	−	+	−	Parietal	−	−	−	−
28	SS	+	+	+	+	+	Mild	Frontal and parietal	Moderate	−	−	−
29	NS	+	−	−	−	+	−	−	−	−	−	−
30	SS	+	+	+	+	+	Severe	Diffuse	Severe	+	−	−
31	NS	+	−	−	+	+	Moderate	Frontal and parietal	Mild	−	−	−
32	SS	−	−	−	−	−	−	−	Mild	−	−	−
33	SS	−	−	−	−	−	−	−	Mild	−	−	−
34	NS	+	−	−	−	−	−	Frontal and parietal	Mild	−	−	−
35	NS	+	+	+	+	+	Severe	Diffuse	Moderate	−	+	−
36	NS	−	−	−	+	+	−	−	Mild	−	−	−
37	SS	−	−	−	+	+	Severe	−	−	−	−	−

In this study, 19 patients were identified by newborn genetic screening for neonatal-based diseases after birth, and 18 patients were identified by blood and genetic screening after exhibiting suspected symptoms; these patients were divided into the NS group and the SS group, respectively. The main imaging findings showed no significant difference between the two subgroups, but the signal change consistent with focal infarct was nearly statistically significant (P = 0.051), as shown in Table 3.

**Table 3:** Differences in MR imaging findings between MMA patients with different clinical subgroups.

MR imaging finding	The group by clinical screening (N, %)		χ^2^	P
	SS (18)	NS(19)		
Cerebral sulcus widened	9 (50.0)	8 (42.1)	0.232	0.746
Cerebellar atrophy	5 (27.8)	2 (10.5)	1.793	0.232
Brainstem thinning	4 (22.2)	1 (5.3)	2.275	0.180
Corpus callosum thinning	8 (44.4)	7 (36.8)	0.222	0.743
Cortical atrophy (sulcal widening)	9 (50.0)	6 (31.6)	1.301	0.325
**Ventricular dilation (0–3 grade)**				
0	12 (66.7)	14 (73.7)	1.128	0.770
Mild	1 (5.6)	2 (10.5)		
Moderate	1 (5.6)	1 (5.3)		
Severe	4 (22.2)	2 (10.5)		
Subcortical white matter change	7 (38.9)	7 (36.8)	0.016	0.898
**Periventricular white matter change**				
0	4 (22.2)	4 (21.1)	1.347	0.718
Mild	12 (66.7)	13 (68.4)		
Moderate	1 (5.6)	2 (10.5)		
Severe	1 (5.6)	0		
Internal capsule change	2 (11.1)	2 (10.5)	0.003	0.954
Signal change consistent with focal infarct	0	3 (15.8)	3.093	0.051
Abnormal signal in basal ganglia	1	0	−	−

**Figure 2:**
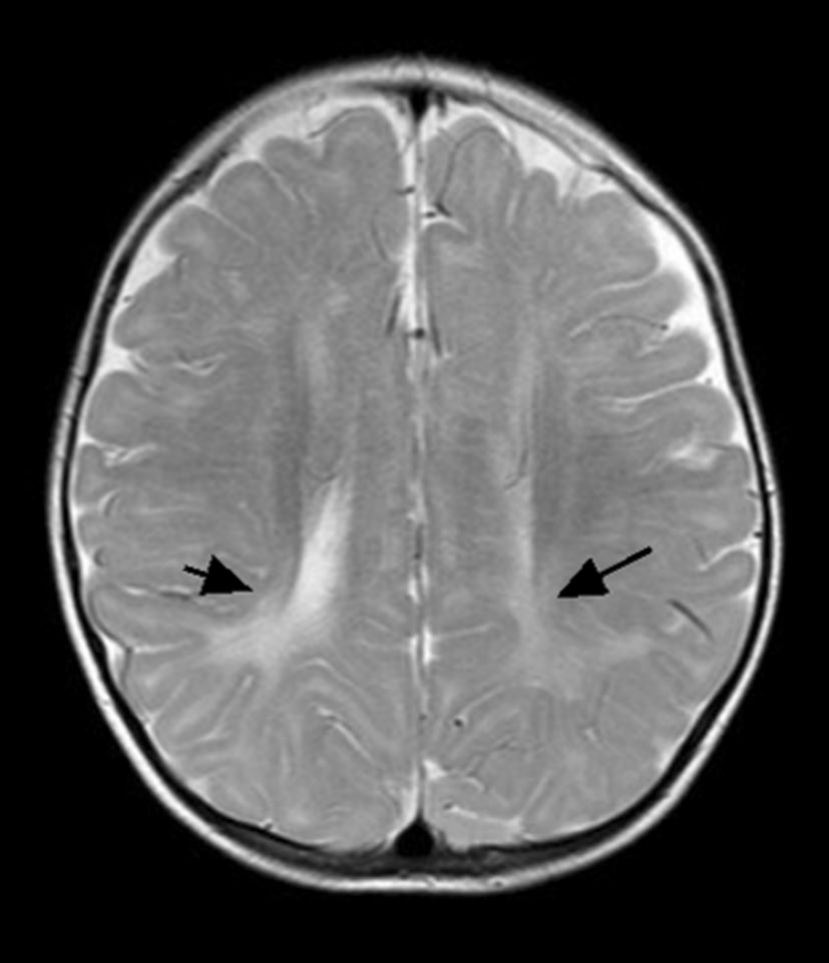
Patient no. 19. axial T2WI showing symmetrical hyperintensity in the matter of the posterior horn of bilateral ventricles.

In this study, 19 patients were identified by newborn genetic screening for neonatal-based diseases after birth, and 18 patients were identified by blood and genetic screening after exhibiting SS; these patients were divided into the NS group and the SS group, respectively. The main imaging findings were not significantly different between the two subgroups, but the signal change consistent with focal infarct was nearly statistically significant (P = 0.051), as shown in Table 3. Regarding sex, significantly more male patients than female patients had subcortical white matter changes (see Table 4, Fig. 2).

**Table 4:** Differences in imaging findings between MMA patients with different gender subgroups.

_MR imaging finding_	_The group by gender (N, %)_		_χ_^2^	_P_
	_Male (27)_	_Female (10)_		
_cerebral sulcus widened_	_14 (51.9)_	_3 (30.0)_	_1.403_	_0.288_
_Cerebellar atrophy_	_5 (18.5)_	_2 (20.0)_	_0.010_	_0.999_
_Brainstem thinning_	_4 (14.8)_	_1 (10.0)_	_0.145_	_0.704_
_Corpus callosum thinning_	_9 (33.3)_	_6 (60.0)_	_2.153_	_0.142_
_Cortical atrophy (sulcal widening)_	_10 (37.0)_	_5 (50.0)_	_0.509_	_0.476_
_**Ventricular dilation (0–3 grade)**_				
_0_	_20 (74.1)_	_6 (60.0)_	_0.922_	_0.820_
_Mild_	_2 (7.4)_	_1 (10.0)_		
_Moderate_	_1 (3.7)_	_1 (10.0)_		
_Severe_	_4 (14.8)_	_2 (20.0)_		
_Subcortical white matter change_	_13 (48.1)_	_1 (10.0)_	_4.515_	_0.034_
_**Periventricular white matter change**_				
_0_	_6 (22.2)_	_2 (20.0)_	_1.456_^a^	_0.885_
_Mild_	_17 (63.0)_	_8 (80.0)_	_1.345_	_0.764_
_Moderate_	_3 (11.1)_	_0_	_–_	_–_
_Severe_	_1 (3.7)_	_0_	_–_	_–_
_Internal capsule change_	_2 (7.4)_	_2 (20.0)_	_1.200_	_0.291_
_Signal change consistent with focal infarct_	_3 (11.1)_	_0_	_1.209_	_0.272_
_Abnormal signal in basal ganglia_	_1_	_0_	_–_	_–_

**Figure 3:**
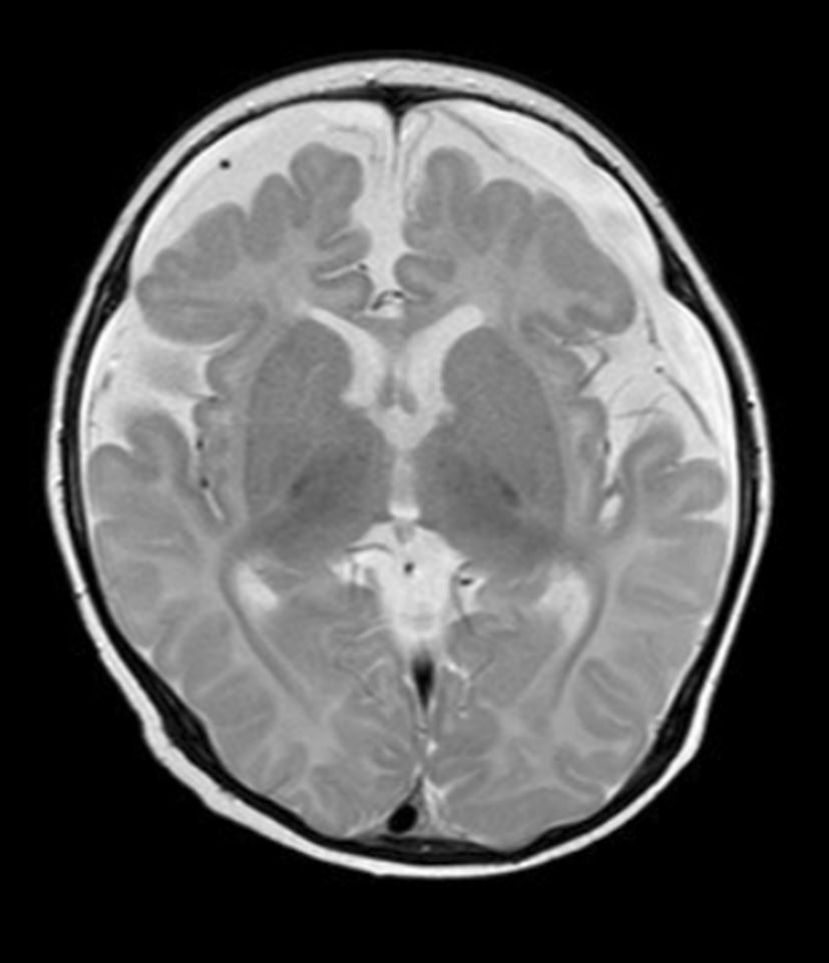
Patient no. 17. axial T2WI showing decreased bilateral frontal lobe volume and widened subarachnoid space.

**Figure 4:**
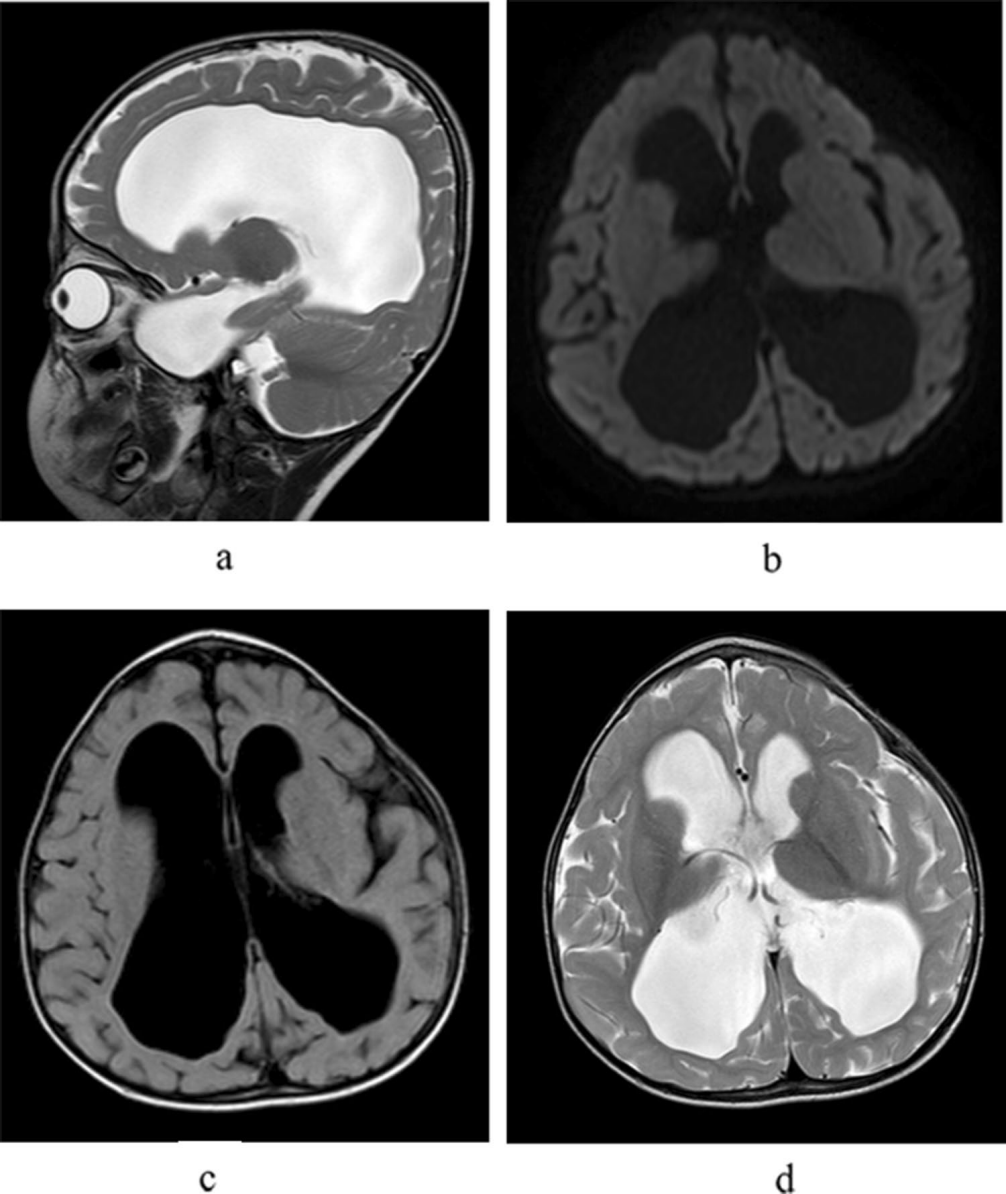
**(a–d)** Patient no. 30. MRI showing severe ventricular dilation and corpus callosal thinning (**a**, sagittal T2WI).

According to developmental quotient, 37 patients were divided into the NI subgroup (developmental quotient ≥ 85) and the LI subgroup (developmental quotient less than 85). The incidence of corpus callosal thinning, cortical atrophy (sulcal widening), subcortical white matter changes, and ventricular dilation (0 to 3 grade) was significantly higher in the LI subgroup than in the NI subgroup (P < 0.05). The incidence of 0-mild and moderate-severe ventricular dilation was significantly higher in the LI subgroup than in the NI subgroup (P < 0.05), as shown in Table 5, Figs. 3 and 4.

**Table 5:** Differences in imaging findings between MMA patients with different developmental quotient subgroups.

MR imaging finding	The group by developmental quotient (N, %)		χ^2^	P
	Normal (11)	Lower (26)		
Cerebral sulcus widened	4 (36.4)	13 (50.0)	0.579	0.447
Cerebellar atrophy	1 (9.1)	6 (23.1)	0.986	0.321
Brainstem thinning	1 (9.1)	4 (15.4)	0.262	0.609
Corpus callosum thinning	0	15 (57.7)	10.673	0.001
Cortical atrophy (sulcal widening)	2 (18.2)	13 (50.0)	3.246	0.052
**Ventricular dilation (0–3 grade)**				
0–mild	11 (100)	18 (69.2)	5.366	0.049^a^
Moderate–severe	0	8 (30.8)	4.318	0.038^a^
Subcortical white matter change	1 (9.1)	13 (50.0)	5.500	0.019
**Periventricular white matter change**				
0	3 (27.3)	5 (19.2)	1.807	0.730^a^
Mild	9 (81.8)	16 (61.5)		
Moderate	0	3 (11.5)		
Severe	0	1 (3.8)		
Internal capsule change	0	4 (15.4)	1.897	0.168
Signal change consistent with focal infarct	1 (9.1)	2 (7.7)	0.020	0.887
Abnormal signal in basal ganglia	0	1 (3.8)	–	

^a^Fisher's exact probability method is used.

Finally, we analyzed the correlation between developmental quotient and ventricular dilation/periventricular white matter changes (grade 0–3) and found that the developmental quotient was significantly negatively correlated with the ventricular dilation grade, as shown in Table 6.

**Table 6:** Correlation analysis between developmental quotient grouping and ventricular dilation/ periventricular white matter change (0–3 grade).

MR imaging finding	Correlation coefficient	P
Ventricular dilation	− 0.546	<0.001
Periventricular white matter change	− 0.096	0.566

## Discussion

MRI has become the preferred imaging examination method for newborns or infants because it uses no radiation and has features including high tissue contrast and high spatial resolution. Thirty-seven children with MMA underwent routine MRI examinations, which can be used to observe delayed myelin development or dysplasia and to evaluate brain development. The most common imaging findings in the MMA patients in this study were subcortical white matter changes (37.8%), periventricular white matter changes (78.4%), ventricular dilation (29.7%) and cerebral atrophy (40.5%). The autopsy report confirmed that neuropathological damage in MMA included encephalatrophy, reactive gliosis, hypomyelination, multifocal cerebellar hemorrhage, and depletion or hypodevelopment of the cerebellar external granule cells in children older than 10 months of age^2,19^. Compared with previous autopsy studies, the MRI findings in this study were similar to those in a previous study.

In 2008, 52 patients were reviewed and reported in a research paper^20^. The MRI findings included ventricular dilation, cortical atrophy, and periventricular white matter abnormalities. Similar to the imaging abnormalities in this study, the incidence rate of these features varies among studies. This may be due to the long time span of previous studies and the use of slightly inconsistent imaging methods. In this study, MRI manifestations were observed in patients at the same medical institution. According to the literature from the past 30 years, the main central nervous system manifestations of MMA are as follows: prominence of the ventricles^21^, myelination abnormalities^22^, cortical atrophy and sulcal widening^23,24^, periventricular white matter abnormalities^25^, internal capsule changes^26^, basal ganglia changes ^27^, cerebellar atrophy, subcortical white matter changes, and thinning of the corpus callosum^28–30^.

Due to the large difference in the number of patients in the isolated MMA (i-MMA) and MMA combined with homocysteinemia (MMA-h) group, no comparisons were made regarding the imaging manifestations. In mainland China, combined MMA and homocysteinemia (cblC type) is the main type of MMA. MMA needs to be diagnosed by laboratory methods, and relatively high concentrations of methylmalonic acid and methyl citrate in patient urine, as determined by GC/MS, can lead to a definitive diagnosis of the disorder. Plasma homocysteine can be measured to identify the gene types involved in MMA^13,31^. Nineteen patients underwent newborn genetic screening for NS group, and 18 patients underwent blood and genetic screening after exhibiting SS group. We found that MMA, as a congenital metabolic disease, caused no significant differences in terms of imaging manifestations between the two groups of patients, whether MMA was found by early neonatal gene screening or MRI examinations after SS were observed. This finding indicates that MRI examination is an essential means of identifying central nervous system damage in MMA patients^5,13^. In this study, there were 27 male and 10 female patients, suggesting that the incidence of the disease may be different between the sexes. Because of the low incidence of the disease and the lack of imaging findings in both sexes for comparison, there were greater white matter changes in male patients with MMA than in female patients with MMA. The sex differences in the clinical manifestations and prognosis of patients should be fully considered.

No standardized assessments of the symptoms or neuropsychological status of patients have been conducted. One difference between this study and previous studies is that the table of neuropsychological development used in the examination of 0- to 6-year-old patients (CNBSR 2016) was applied in all 37 pediatric patients when MRI examinations were performed. The development quotient was calculated to evaluate the neuropsychological development status of the MMA patients (Table 1). The incidence of corpus callosal thinning, cortical atrophy, and ventricular dilation on imaging findings was higher in the LI subgroup than in the NI subgroup. Abnormal MRI findings provide a visual basis for understanding morphological changes in the impaired neuropsychological development of MMA patients. It is suggested that the correlation between the damage to the central nervous system and the neuropsychological developmental state of children with MMA can be evaluated based on MRI findings to objectively evaluate the curative effect of standard treatments.

There were 11 patients with ventricular dilation, of whom 8 children showed moderate to severe mental retardation, suggesting that ventricular dilation is an important imaging feature of neuropsychological developmental disorders in children with MMA. Previous studies in the literature have reported that ventricular dilatation is also common in MMA patients, and some scholars have suggested that its formation mechanism might be related to intracerebral vascular stiffness, causing the pliancy of arteries to decrease, arterial pressure to increase, arterial delivery pressure to remain the same, and intracerebral pressure to increase and leading to hydrocephalus^32^. The toxicity of high concentrations of cysteine metabolites to the vascular wall is the main cause of vascular endothelial damage^33^. In our study, follow-up imaging findings of MMA patients after treatment were lacking; these images could help to explain the correlation between changes in the degree of ventricular dilatation and neuropsychological scores after treatment. Longitudinal research with more than one MRI scan from each patient will be indispensable to verify this hypothesis.

We observed cerebral sulcal widening and cerebellar atrophy and found that the LI group had a markedly higher incidence of cortical atrophy (sulcal widening) than the NS group. According to previous reports, the most common imaging manifestation of MMA is cerebral atrophy^20^, which is characterized by different degrees of cerebral and cerebellar sulcal deepening, cortical atrophy, and widening of the extracerebral space. The cause of brain shrinkage is actually a reduction in the volume of the white matter, which is known as stunted growth. Cerebral atrophy can directly affect the development of the cerebral nervous system in newborns or infants. Diffuse supratentorial white matter edema, which was reported in an Italian study^34^, was not observed in our patients. In spite of reports of cerebellar hemorrhage in metabolic disorders^35,36^, there were no MRI findings of this complication in these patients. In this study, conventional MRI sequences were performed,however, susceptibility-weighted sequences that can more sensitively detect hemorrhage were not performed. Moreover, there were seven patients with cerebellar atrophy that could have conceivably resulted from cerebellar lesions. However, such findings were not significantly different among the different groups. There have been recent reports of hemorrhagic and necrotic foci in the brainstem as well as hypomyelination and spongy changes in the brainstem nuclei^19^. Five children showed brainstem atrophy, and four children showed internal capsular changes in this study; these incidence rates are higher than those observed in previous studies^20^.

The abnormal white matter changes in MMA patients are usually located in the anterior and posterior corners of both lateral ventricles and in the centrum semiovale with a symmetrical distribution. Mild delays in myelination can be difficult to identify on cerebral MRI; T2WI hyperintensity around the ventricles and subcortical white matter T2WI hyperintensity were observed. The incidence of subcortical white matter hyperintensity was significantly higher in the SS group (50%) than in the NS group (9%). The mechanism driving this result may be related to the brain S-adenosine methionine deficiency caused by metabolic abnormalities^37^. No children older than 1 year showed this manifestation, which is consistent with the results of a study by Brismar and Ozand showing that MMA leads to some delays in myelination rather than an “absent development”^21^. If myelin sheath abnormalities cannot be diagnosed and effectively treated at an early stage, the volume of white matter will decrease and lead to a poor prognosis. Corpus callosal thinning, which was reported by Enns et al.^24^, is common in patients with white matter changes, either acquired or hereditary. Abnormal MRI signals and corpus callosum thinning in bilateral ventricles and the periventricular white matter in the centrum semiovale may be related to myelin dysplasia or developmental delays^38^. Hypomyelination and delayed myelination have been found in patients with MMA. It is tempting to assume that callosal atrophy is a reflection of prevalent abnormal white matter changes in MMA patients.

The basal ganglia (especially the pallidum) were the most frequently affected areas in MMA patients. The formation mechanism may be related to the decreased activity of cytochrome C oxidase and succinic acid dehydrogenase, resulting in the accumulation of toxic organic acid metabolites and leading to damage to the basal ganglia with high energy requirements, especially the globus pallidus^39–41^. According to previous research, the basal ganglia were affected in MMA patients^21,22,25,26^. Both necrosis and hemorrhage have been reported. In the medical imaging literature, calcification of the basal ganglia has been repeatedly mentioned. Calcification could be the end result of either hemorrhage or necrosis^27,28^. In the acute attack stage of MMA, the globus pallidus showed low density on computed tomography (CT) images. MRI showed hypointensity on T1WI, hyperintensity on T2WI, hyperintensity on DWI, hypointensity on apparent diffusion coefficient (ADC) imaging and a symmetrical distribution. However, in our study, no abnormal signals were found in the globus pallidus, and a large sample of cases should be further examined. In our study, one patient (no. 19) presented symmetrical T1WI hyperintensity in the caudate nucleus, which showed DWI hyperintensity and corresponding ADC hypointensity (Fig. 1). The patient was diagnosed with bleeding by MRI findings, accompanied by severe ventricular dilatation, corpus callosal thinning, and white matter changes, with a developmental quotient of only 69.6. This patient should also be studied longitudinally to understand the relationship between changes in imaging characteristics and changes in the neuropsychological development scale through follow-up observation.

One strength of this study is that all patients underwent MRI examinations using the same MRI equipment at the same medical institution with the exact same scanning parameters. In future studies, we may consider incorporating quantitative susceptibility maps (QSM) into the diagnosis of basal ganglia lesions. It is possible that changes in the globus pallidus do not appear until advanced stages of the disease^42^. All patients underwent MRI examinations after neonatal genetic screening or after the appearance of SS of MMA. The examination period was early, which may be why no calcification was observed in the basal ganglia. Furthermore, considering the image thickness and the slice gap used in our MRI protocol, it is possible that tiny abnormalities in the basal ganglia were omitted.

Some limitations of our study also have to be mentioned. We did not explored the relationship between MRI abnormalities and mutation data or clinical metabolic that will be further elucidated in subsequent studies, and not fully considered consequences of an inborn error of metabolism (IEM) or effects due to delayed diagnosis and possible medical mismanagement. This study was a preliminary cross-sectional design study of brain manifestations MRI findings in patients with MMA, which is part of our overall IEM study. Furthermore, we continue to expand the sample size and the observations of the relationships between i-MMA and MMA-h patient imaging characteristics and neuropsychological scores. We will also apply multi-modal MRI [the brain structure of 3D T1WI, QSM, blood oxygen level-dependent (BOLD), magnetic resonance spectroscopy (MRS) etc.] in these studies to obtain additional morphological and functional data, and the relevant results will be published in subsequent studies. No follow-up data is a limitation of this study. The clinicians look forward to understanding the improvement of neuropsychological scores of patients with MMA. Parents are very eager to know the rehabilitation status of the patients to understand the changing trends of their conditions, which is important for the patients and their parents to establish confidence in the next treatment. In future studies, we should continue to follow up with the patients included in this study for many years to obtain neuropsychological scores and MRI data at multiple time points.

## Conclusion

Ventricular dilatation, cerebral atrophy, white matter changes and corpus callosal thinning are the main MRI abnormalities in MMA patients, and these manifestations are significantly correlated with delayed development in children. The presence of relevant clinical and MRI findings should raise a high degree of suspicion of MMA in pediatric radiologists and pediatricians. MRI findings can be considered an important basis for determining the severity of MMA in individual patients and assessing prognosis.

## Supplementary information

Supplementary Information.
